# An Observational Study on the Application of the Second-generation Radiopaque Subcutaneous Implant

**DOI:** 10.22037/ijpr.2020.114527.14892

**Published:** 2021

**Authors:** Xiaoou Xu, Xiaojuan Zhang, Xueniu Yang

**Affiliations:** a *NHC Key Laboratory of Birth Defects and Reproductive Health (Chongqing Key Laboratory of Birth Defects and Reproductive Health, Chongqing Population and Family Planning Science and Technology Research Institute), Chongqing 400020, China. *; b *Department of Clinical Medicine, Chongqing Medical and Pharmaceutical College, Chongqing, China.*

**Keywords:** Subcutaneous implantation, Contraception, Radiopaque, Side effects

## Abstract

To analyze the effect, side effects, and satisfaction of the second-generation radiopaque subcutaneous implant, providing healthcare workers a basis for propaganda and guidance. Methods: A total of 214 cases of healthy women with birth control requirements from August 2016 to June 2018 received ray subcutaneous implants. Quantitative surveys were administered 10 days, 3 months, 12 months, and 24 months later. All cases were implanted successfully. The number of effective cases was 211, with 3 cases lost to follow-up. The pregnancy rate of 211 subjects during receiving implant was 0. Changes in bleeding patterns (130 cases, 61.61%) remained the most common side effect event. The rate of withdrawal was 96.21% in the first year and 66.35% in the second year. There were 48 cases (67.61%) who withdrew due to the change of bleeding pattern. All participants with bleeding patterns were divided into the withdrawal group and the non-withdrawal group, and there were significant differences in educational background, number of pregnancies, abortion, and income between the two groups. According to the satisfaction survey, 37 (17.54%) were very satisfied, 109 (51.66%) were satisfied. The contraceptive rate of radiopaque subcutaneous implantation was very high, and there were no serious side effects while receiving an implant. The change of bleeding pattern was the main reason for withdrawal, which was related to educational background, number of pregnancies, history of abortion, and economic conditions. Pre-implantation consultation and post-implantation management should be strengthened.

## Introduction

Subcutaneous implantation is one of the long-acting contraceptive methods, and it is also recognized as one of the effective contraceptive methods with excellent performance in the world. Clinical studies on subcutaneous implantation of contraceptive drugs began in the 1960s (1). After nearly 50 years of development, a single subcutaneous embedding agent has been more convenient for women to use and take out (2-4). Although subcutaneous implants have been approved in more than 60 countries worldwide, millions of women use them. However, in China, condoms, IUDs/systems and oral contraceptives are still the preferred methods of birth control, and the using rate of subcutaneous implantation is less than 10%. Our previous investigation found that less than 5% of induced abortion women in Chongqing would choose subcutaneous embedding agents (5). The main reason is the lack of knowledge about Implanon and worrying about its possible side effects.

In July 2016, the second-generation radiopaque etonogestre implant was used in the affiliated hospital of Chongqing Science and Technology Research Institutein ahead of all hospitals in Chongqing, China. At present, compared with the European and American countries, there are few studies on etonogestrel subcutaneous implants in China, and there are no reports on the efficacy and safety of impenetrable radiation subcutaneous implants in China, and there are also few reports on the reasons why users of subcutaneous implants quit midway in China (6-8). This study reviewed 214 cases of radiopaque subcutaneous implants user on effectiveness, safety and the reasons for withdrew, and aimed to provide people more data for the efficacy and safety of the second generation radiopaque subcutaneous implants. At the same time, it would not only provide healthcare workers a basis for propaganda and guidance but also benefit the continuous improvement of new subcutaneous implants through investigating the user’s acceptability of radiopaque subcutaneous implanon.

## Experimental

*Subjects*

214 healthy women with contraceptive needs were admitted to the Affiliated Hospital of Chongqing Population and Family Planning Science and Technology Research Institute from August 2016 to June 2018. All participants had no history of subcutaneous implantation.

Inclusion criteria: In need of long-term contraception; unsuitable (*e.g*., genital deformity) and unwilling to place IUD; failure to place IUDs for many times; those who need long-term contraception and are not suitable for sterilization or worry about sterilization; those who were allergy to the birth control pill containing estrogen, or who are difficult to continue to use the birth control pill.

Exclusion criteria: pregnant or suspicious pregnant, unwilling to use subcutaneous implant; active venous thromboembolism disease, severe liver disease, known or suspected sex hormone-sensitive malignant tumor, current liver tumor or history of liver tumor (benign or malignant), unexplained vaginal bleeding, allergy to any component of the product.

*Data collection*

A questionnaire was developed by referring to relevant research results reported, and a telephone follow-up survey was conducted by specially trained interviewers (family planning physicians) according to the unified questionnaire. The participants were followed up on the 10th day, 3rd, 6th, 12th and 24th month after implantation of impenetrable radiation subcutaneous implant (etonogestrel) on the 1st to fifth day after menstruation or after artificial abortion. Symptoms, clinical manifestations, changes in bleeding patterns and adverse events were recorded at each follow-up visit. The user’s satisfaction survey was conducted at the visit was terminated.

Data were collected as follows: 1) name, age, education background, economic income; 2) Menstrual conditions before subcutaneous implantation: age of first menstruation, menstrual cycle, menstrual period, menstrual volume, dysmenorrhea. 3) Fertility history: number of pregnancies, number of births, number of abortions. 4) Contraceptive methods 3 months before implantation. 5) side effects after subcutaneous implantation: menstrual changes, weight changes, facial acne, ovarian cysts, early redness and swelling of the subcutaneous implantation site, pain, hormonal contraception-related adverse reactions, etc. 6) Others: the use of subcutaneous implantation, user satisfaction, whether to recommend others to use.

*Data analysis /ethical issues*

All data were analyzed in SPSS 20.0. Cases and percentages expressed enumeration data. All tests were two-sided, and the level of significance was set at *p* < 0.05.

This study obtained ethical approval from Chongqing Population and Family Planning Science and Technology Research Institute, and its conduct was guided by guidelines on the conduct of research in human subjects. All participants agreed to enter this study, and the authors have no conflict of interest to declare. 

## Results

*Demographic information*

Of 214 women enrolled in this study, 3 cases (1.4%) were lost to follow-up, resulting 211 cases for inclusion in the analysis. The majority of women aged 30-39 years old and only 2 patients aged 19 years old or below (0.95%). All participants had a pregnancy history, including 155 cases with 3 or more pregnancies, accounting for 73.46%. In the 3 months before implantation, there were 109 (51.66%) cases that used condoms as a contraceptive method. 206 (97.63%) had a birth history. 111 (62.61%) participants had college degrees, while those who had bachelor’s degrees and above were the least (10.90%). 196 (92.89%) had a history of abortion in the past. There were 107 (50.71%) whose monthly income was between 2000 and 5000 RMB. See Table 1.

*Contraceptive effect*

Successful rate of subcutaneous implantation of 211 subjects was 100%. The pregnancy rate during placement was 0, and the contraceptive rate was 100%.

*Adverse events*

The changes in the bleeding pattern was still the most common adverse event, with 39 (18.48%) endless menstruation, 23 (10.90%) irregular menstruation and prolonged menstruation, 17 (8.06%) irregular menstruation and increased menstrual volume, 19 (0.90%) irregular menstruation and decreased menstrual volume, 16 (7.58%) amenorrhea, and 16 (7.58%) frequent menstruation. Besides bleeding patterns change, there were 9 (4.27%) weight gain cases, 13 (6.12%) dizziness and headache cases and 4 (1.90%) acne cases. In addition, we found that 1 case had consciously low sexual desire drops and 1 had flustered and sleep quality drop as well, as 1 case’s upper lip hair increased. Other effects were found, including blood pressure significantly decreased in 1 case and the right ovarian cyst in 1 case. After implantation, there was no abnormal blood count, coagulation function, and liver and kidney function. See Table 2.

*Acceptability*

After 1 year of placement, 8 cases withdrew, and the continuance using rate was 96.21%. Among them, 4 withdrew due to changes in bleeding pattern, 1 withdrew due to divorce without contraception, 2 withdrew due to family planning requirements, and 1 withdrew due to pain and discomfort in the left lower extremity on the second day after placement (after examination, it was confirmed that the implantation was unrelated, and the patient was diagnosed as acute osteomyelitis. After the removal, due to the failure of contraception again, he came to our hospital to place the intrauterine device containing copper

After 2 years of treatment, 63 cases were withdrawn, and the rate of continuation using was 66.35%. Bleeding patterns change out of 44 cases with birth plan calls for exit 11 cases, dizziness headache exit in 2 cases, severe acne exit 1 case, obviously increase the weight exit in 2 cases, consciously low sexual desire with bleeding patterns change sex amenorrhoea (drugs) out of 1 case, consciously flustered sleep quality decline associated with bleeding pattern change exits in 1 case, upper lip hair consciously increase in 1 case. See Table 3 for details.

*Comparison of demographic character-istics between the withdrawal group and the non-withdrawal group due to changes in bleeding pattern*

During the follow-up period, 130 cases had bleeding patterns changed. The demographic characteristics analysis of the withdrawal group and the non-withdrawal group showed significant differences in the number of pregnancies, previous history of abortion, educational background and economic income between the two groups (*p* < 0.05). The withdrawal rate was highest for those with one pregnancy (70.59%), while for those with two, three or more pregnancies, it was 61.11% and 26.31%, respectively. As far as the educational background is concerned, the withdrawal rate was 37.14% for those with a high school degree or below, 29.87% for those with college, 66.67% for bachelor’s degree and above. The withdrawal rate of those with abortion was 33.90%, while that of those without abortion was 66.67%. The withdrawal rate for those with a monthly income of 2000 and below was 58.33%, 24.44% for those with a monthly income of 2000 to 5000, and 67.86% for those with a monthly income of 5000 and above. However, there were no significant differences in age and contraceptive methods in 3 months before implantation (*p *> 0.05). See Table 4.

According to the satisfaction survey, 37 cases (17.54%) were very satisfied, and 109(51.66%) were satisfied, while 48 (22.75%) were general, 16 (7.58%) were dissatisfied, and 1 (0.47%) very dissatisfied. Meanwhile, 53 (25.12%) indicated that they would definitely recommend to others, 99 (46.92%) might recommend, and 34 (16.11%) not sure, while 25 (11.85%) indicated that they would not recommend and 0 indicated that they would definitely not recommend, as shown in Table 5.

## Discussion

In this observational study, the demographic characteristics of the users showed that the majority of people aged 20-29 years old, only 2 participants under 19 years old, accounting for the least proportion. The majority had more than 3 pregnancies, and the vast majority had a history of childbearing, while those who had not had children used very little. Although the unmarried and childless populations and adolescents are the applicable objects of subcutaneous implantation, the actual use rate is very low. The low using rate among those populations is possibly related to the lack of knowledge and the long-term domestic publicity and education related to reproductive health. It is partly different from the demographic characteristics of participants reported by Qin Taizhou *et al.* (7). Our research found that a college degree accounts for a relatively higher proportion than those of Qin’s report, which is possible due to the different research time and different areas. As we all know, the overall level of education of the population in China has been improved. Meanwhile, we also found that those with higher income chose to embed agents for a higher proportion, which may be because embedding agents need cost a sum of money, and people can afford the Implanon if they had a relatively higher income.

This study used the second generation of radiopaque embedding agents, which was ahead of many hospitals in China. In the follow-up period, there was no pregnancy, and the contraceptive rate was 100%. Its good contraceptive effect was similar to that of the first generation of radiopaque implants and non-radiopaque implants. The use of auxiliary material barium sulfate increased the function of radiopaque, and its main purpose was to facilitate tracking and positioning after placement. In the risk assessment of barium sulfate, there were no safety problems related to barium sulfate. At the same time, barium sulfate did not affect the function of the drug, and the contraceptive effect was not affected. Meanwhile, although the radiopaque function was added to track the implant’s displacement, there was no ectopic displacement or subcutaneous migration during the implantation and follow-up period, so an X-ray was not used to find the implant. This is related to the operator’s skilled technique and the patient’s subcutaneous tissue condition, as well as to the good product design.

In addition, we observed the adverse events and found that the side effects were similar to the previous non-radiopaque implants. There were mainly bleeding pattern changes, acne, weight gain, and dizziness and headache (9-12). Among them, the change of bleeding pattern is still the main adverse event. No abnormal blood count, liver and kidney function, coagulation function and other serious adverse events were found.

According to the acceptability, the one-year continuous use rate was 96.21%, which was significantly higher than that previously reported in China (13,14). The two-year continuous using rate was 66.35%, similar to that reported abroad (15-17) (there were few reports on the 2-year continuous using rate in China). In this study, 8 cases quit from the survey in the first year. The main reasons for withdrawal were the change of menstrual pattern, other adverse reactions, and fertility requirements. In the second year of follow-up, 63 cases withdrew from the study, and the main reason was still the change of bleeding mode, which was similar to the related reports (18). Further analysis of demographic characteristics showed that those with fewer pregnancies, no history of abortion, bachelor’s degree or above, and higher income were more likely to quit. However, there was no significant difference in age and contraceptive methods in 3 months before implantation between the two groups, suggesting that previous pregnancy history, abortion, education background and income had an impact on the acceptability of subcutaneous implants.

Satisfaction survey shows that 37 cases (17.54%) were very satisfied, 109 cases (51.66%) were satisfied, 48 cases (22.75%) were not satisfied, which was similar to those of reports from domestic and abroad (7,18). In addition, it also shows that 53 (25.12%) would definitely recommend to others and 99 (46.92%) would probably recommend, but 25 (11.85%) would probably not recommend. This survey suggests that with the increasing use of a subcutaneous implant, their acceptability became higher and higher.

**Table 1:** Demographic characteristics of participants

**age** **（** **year** **）**	**n** **（** **%)**
≤19	2 (0.95%)
20-29	78 (36.97%)
30-39	114 (54.03%)
40-45	17 (8.06%)
**pregnancy times**	
0	0 (0%)
1	29 (13.74%)
2	27 (12.80%)
≥3	155 (73.46%)
**the contraceptive method in the 3 months before implantation**	
Oral bills	13 (6.16%)
IUD	31 (14.69%)
Condom	109 (51.66%)
No contraception	56 (26.54%)
other	2 (0.95%)
**Birth history**	
Yes	206 (97.63%)
No	5 (2.37%)
**Educational background**	
High school and below	77 (36.49%)
college	111 (62.61%)
bachelor degree and above	23 (10.90%)
**Abortion history**	
yes	196 (92.89%)
no	15 (7.11%)
**Monthly income**	
<2000	21 (9.95%)
2000-5000	107 (50.71%)
>5000	86 (40.76%)

**Table 2:** Number of cases of side effects and proportion in total participants

		**10 days**		**3 months**		**6 months**		**12 months**		**24 months**		**Total**	
		**n** **（** **%** **）**		**n** **（** **%** **）**		**n** **（** **%** **）**		**n** **（** **%** **）**		**n** **（** **%** **）**		**n** **（** **%** **）**	
endless		0 (0%)		5 (2.37%)		6 (2.84%)		12 (5.69%)		16 (7.58%)		39 (18.48%)	
prolonged		0 (0%)		2 (0.95%)		5 (2.37%)		9 (4.27%)		7 (3.31%)		23 (10.90%)	
Increased volume		0 (0%)		3 (1.425)		4 (1.90%)		5 (2.37%)		5 (2.37%)		17 (8.06%)	
decreased volume		0 (0%)		3 (1.42%)		9 (4.27%)		3 (1.42%)		4 (1.90%)		19 (0.90%)	
amenorrhea		0 (0%)		4 (1.90%)		4 (1.90%)		4 (1.90%)		4 (1.90%)		16 (7.58%)	
frequent		0 (0%)		4 (1.90%)		4 (1.90%)		4 (1.90%)		4 (1.90%)		16 (7.58%)	
weight gain		0 (0%)		1 (0.47%)		1 (0.47%)		3 (1.42%)		4 (1.90%）		9 (4.27%)	
Dizziness\ headache		2 (0.95%)		3 (1.42%)		4 (1.90%)		2 (0.95%)		2 (0.95%)		13 (6.12%)	
acne		0 (0%)		1 (0.47%)		2 (0.94%)		1 (0.47%)		1 (0.47%)		4 (1.90%)	
Low sexual desire		0 (0%)		1 (0.47%)		1 (0.47%)		0 (0%)		1 (0.47%)		3 (1.42%)	
sleep quality drop		1 (0.47%)		1 (0.47%)		1 (0.47%)		1 (0.47%)		1 (0.47%)		5 (2.37%)	
Upper lip hair increased		0 (0%)		1 (0.47%)		1 (0.47%)		1 (0.47%)		1 (0.47%)		4 (1.90%)	
blood pressure decreased		0 (0%)		1 (0.47%)		1 (0.47%)		1 (0.47%)		1 (0.47%)		4 (1.90%)	
ovarian cyst		0 (0%)		0 (0%)		0 (0%)		1 (0.47%)		0 (0%)		1 (0.47%)	

**Table 3:** Number and proportion of withdrawal from follow-up for different reasons

	**The 1st year (n = 211** **）** **n (%)**	**The 2nd year (n = 203)** **n (%)**
changes in bleeding pattern	4 (1.90%)	44 (21.67%)
family planning requirements	2 (0.95%)	11 (5.42%)
dizziness and headache	0 (0%)	2 (0.99%)
acne	0 (0%)	1 (0.49%）
Weight gain	0 (0%)	2 (0.99%)
pain and discomfort in the left lower extremity	1 (0.047%)	1 (0.49%)
divorce without contraception	1 (0.47%)	0 (0%)
low sexual desire	0 (0%)	1 (0.49%)
flustered and sleep quality drop	0 (0%)	1 (0.49%)
upper lip hair increased	0 (0%)	1 (0.49%)

**Table 4:** Comparison of demographic characteristics between the withdrawal group and the non-withdrawal group due to the change of bleeding pattern

	**Withdrawal** **group n** **（** **%)**	**non-withdrawal** **group n** **（** **%)**	**Chi-** **value**	***p*** **-value**
**age** **（** **year** **）**				
≤19	1	1		
20-29	17	23		
30-39	25	51		
40-45	5	7	1.326	0.723
**pregnancy times**				
1	12	5		
2	11	7		
≥3	25	70	17.384	<0.001
**the contraceptive method in the 3 months before implantation**				
Oral bills	4	3		
IUD	8	10		
Condom	31	62		
No contraception	5	7	2.297	0.513
**Educational background**				
High school and below	13	22		
college	23	54		
bachelor degree and above	12	6	8.483	0.014
**Abortion history**				
yes	40	78		
no	8	4	5.022	0.032
**Monthly income**				
<2000	7	5		
2000-5000	22	68		
>5000	19	9	19.88	<0.001

**Table 5:** Satisfaction of participants and whether they will recommend an implant to others

	**n(%** **）**
**Satisfaction**	
very satisfied	37 (17.54%)
satisfied	109 (51.66%)
general	48 (22.75%)
dissatisfied	16 (7.58%)
very dissatisfied	1 (0.47%)
**Recommended to others**	
sure	53 (25.12%)
possibly	99 (46.92%)
unsure	34 (16.11%)
may not	25 (11.85%)
absolutely not	0 (0%)

## Conclusion

The contraceptive rate of the radiopaque subcutaneous implant is very high, and there is no serious adverse event. However, the trouble caused by the change of bleeding mode is still the most common side effect. The results show that the first year of the continuous using rate of the radiopaque subcutaneous implant is higher than that reported in the literature. The change of bleeding mode is the main reason for users to quit. The number of pregnancies, abortion history, education background, and economic income significantly influence the withdrawal due to the change of bleeding mode. Therefore, it is the key to improve the acceptability and the rate of continuous use of the implant through strengthening the pre-implantation consultation and post-implantation management, eliminating the psychological burden of the participants, and finding the reasons and treatment methods of adverse reactions caused by the implant.

## Authors’ contributions

XX was the Principal Investigator for the study and designed the study, trained the data collectors, supervised data collection. ZXJ conducted the main analysis and wrote the main portions of this manuscript. YXN contributed to the data collectors training, study supervision, statistical analysis and reviewed and edited this manuscript. All authors read and approved the final manuscript. 

## Funding

This work was supported by the Scientific research project of Chongqing Health Commission [grant number: 2017MSXM068 and 2017MSXM064]. 

## Availability of data and materials

The datasets generated and analyzed during the current study are not publicly available due to their continued use in ongoing research but are available from the corresponding author on reasonable request. 

## Ethics approval and consent to participate

Data collection for this research was approved by Chongqing Population and Family Planning Science and Technology Research Institutional Review Board, as well as by the Ethics Committee 

of the Chongqing Population and Family Planning Science and Technology Research Institute. All participants provided written informed consent to participate in the study. 
